# Samango Monkeys (*Cercopithecus albogularis labiatus*) Manage Risk in a Highly Seasonal, Human-Modified Landscape in Amathole Mountains, South Africa

**DOI:** 10.1007/s10764-016-9913-1

**Published:** 2016-08-19

**Authors:** Katarzyna Nowak, Kirsten Wimberger, Shane A. Richards, Russell A. Hill, Aliza le Roux

**Affiliations:** 1 Evolutionary Anthropology Research Group, Durham University, Durham, DH1 3LE UK; 2 Zoology and Entomology, University of the Free State, Qwaqwa Campus, Phuthaditjhaba, 9866 South Africa; 3 Biological Sciences, University of Cape Town, Rondebosch 7701, Cape Town, South Africa; 4 Commonwealth Scientific and Industrial Research Organisation, Canberra, ACT 2600 Australia

**Keywords:** *Cercopithecus mitis*, Giving-up density, Human disturbance, Landscape of Fear, Guenon

## Abstract

Wild species use habitats that vary in risk across space and time. This risk can derive from natural predators and also from direct and indirect human pressures. A starving forager will often take risks that a less hungry forager would not. At a highly seasonal and human-modified site, we predicted that arboreal samango monkeys (*Cercopithecus albogularis labiatus*) would show highly flexible, responsive, risk-sensitive foraging. We first determined how monkeys use horizontal and vertical space across seasons to evaluate if high-risk decisions (use of gardens and ground) changed with season, a proxy for starvation risk. Then, during a subsequent winter, we offered equal feeding opportunities (in the form of high-value, raw peanuts) in both gardens and forest to see if this short-term change in food availability and starvation risk affected monkeys’ foraging decisions. We found that during the food-scarce winter, monkeys foraged outside indigenous forest and in gardens, where they fed on exotic species, especially fallen acorns (*Quercus* spp.), despite potential threats from humans. Nevertheless, and as predicted, when given the choice of foraging on high-value foods in gardens vs. forest during our artificial foraging experiment, monkeys showed a preference for a safer forest habitat. Our experiment also indicated monkeys’ sensitivity to risk in the lower vertical strata of both habitats, despite their previous extensive use of the ground. Our findings support one of the central tenets of optimal foraging theory: that risk of starvation and sensitivity to the variation in food availability can be as important drivers of behavior as risk of predation.

**Electronic supplementary material:**

The online version of this article (doi:10.1007/s10764-016-9913-1) contains supplementary material, which is available to authorized users.

## Introduction

Animals do not use landscapes equally across time and space, with their movements generally influenced by a combination of food availability, habitat features, and predation risk (Coleman and Hill 2014; Druce *et al*. 2009; Makin *et al*. 2012; Stears and Shrader 2015; Willems and Hill 2009). A food patch is chosen on the basis of a trade-off between feeding rate and predation risk (Brown 1999). Although areas of high predator density or human disturbance are generally avoided (Abu Baker *et al*. 2015; Brown and Kotler 2004; Makin *et al*. 2012), a starving forager will often take risks that a less hungry forager would not, based on the economic calculation that certain death by starvation is more risky than possible death from predation (Brown and Kotler 2007; Dill and Fraser 1984).

People profoundly affect the ways in which wild animals assess risk (Coleman *et al*. 2008; Nowak *et al*. 2014) and distribute themselves across space (Blumstein 2014; Frid and Dill 2002; Tadesse and Kotler 2012). For example, the effects of humans on the foraging and vigilance behavior of elk (*Cervus elephus*) were found to surpass those of both natural predators and habitat type (Ciuti *et al*. 2012). Likewise, Nubian ibex (*Capra nubiana*) left more food uneaten at artificial foraging stations during weekends when human visitation to a national park was high, suggesting that ibex respond to humans as they would to a predator (Tadesse and Kotler 2012). Contrarily, opportunistic mammals such as baboons (*Papio* spp.) may be attracted to human-occupied areas because of the potential resources they offer (Hoffman and O’Riain 2011; Strum 2010) or the safety from natural predators they confer (Berger 2007). Such risky behavior can be motivated by the scarcity of wild fruits (Hockings *et al*. 2009; Wimberger *et al. in prep*.); for example, chimpanzees (*Pan troglodytes*) in Bossou, Guinea, take risks to consume cultivars, especially sugar fruits at certain times of year (Hockings and McLennan 2012). The strength of an animal’s behavioral response to human presence is patently related to its condition (Beale and Monaghan 2004) and, as thirst or hunger and risk of starvation increase, animals will select more hazardous foraging sites and engage in riskier behavior (Sih 1980; Verdolin 2006).

The relative riskiness of an area can be quantified in both time and space. Artificial foraging experiments in the form of giving-up densities (GUDs) help estimate the point at which an animal stops foraging as the risk of predation and lost opportunity costs outweigh energetic gains (Brown 1988). GUDs have been effectively used to gauge the perceived risk and habitat preferences of rodents (Brown 1988), ungulates (Stears and Shrader 2015; Tadesse and Kotler 2012), and primates (Emerson and Brown 2013; Emerson *et al*. 2011; Makin *et al*. 2012; Nowak *et al*. 2014). This technique allows researchers to go beyond the binary classification of “high-risk” and “low-risk” areas, highlight the relative degree of risk faced in different parts of an animal’s microhabitat, and take seasonal changes into account as well.

We aimed to examine how a group of arboreal monkeys perceives the threat imposed by humans and human infrastructure when food availability is seasonally low at the southern limit of their range in Hogsback, Eastern Cape, South Africa (Lawes 1990). Samango monkeys (*Cercopithecus albogularis labiatus*: Dalton *et al*. 2015)) are endemic to South Africa, where they are Red-Listed as Vulnerable (Linden *et al*. 2016), having declined by >30 % in the past *ca*. 30 yrs and now confined to remaining forest fragments (Lawes 2008). At Hogsback, samango monkeys inhabit a human-modified habitat in which they frequent a village and gardens to feed on the seeds of exotic oaks (*Quercus* spp.) and black wattle (*Acacia* sp.) (Wimberger *et al*., *in prep*.) where humans (who chase and shoot monkeys) and domestic dogs (which chase and bite monkeys) pose the major threats to monkeys. Using behavioral data, we first examined how monkeys use horizontal space (residential gardens vs. indigenous Afromontane forest) and vertical space (ground vs. tree level) across four distinct seasons. In this way we evaluated if high-risk decisions (use of gardens and ground) changed with season, a proxy for starvation risk. Few researchers get the opportunity to change this economic calculation for their study subjects. During a subsequent winter, we offered equal feeding opportunities in both gardens and forest to assess monkeys’ relative perceived risk and patch use with a GUD experiment. We predicted that 1) arboreal monkeys perceive gardens and the ground to be riskier than indigenous forest and the tree canopy; 2) monkeys will use gardens and the ground more extensively during winter, when forest food availability is relatively lower; and 3) given equal feeding opportunities in both habitats (gardens and forest) during winter, monkeys will demonstrate a flexible, opportunistic foraging strategy and show a preference for the less risky indigenous forest.

## Methods

### Study Site

**Fig. 1:**
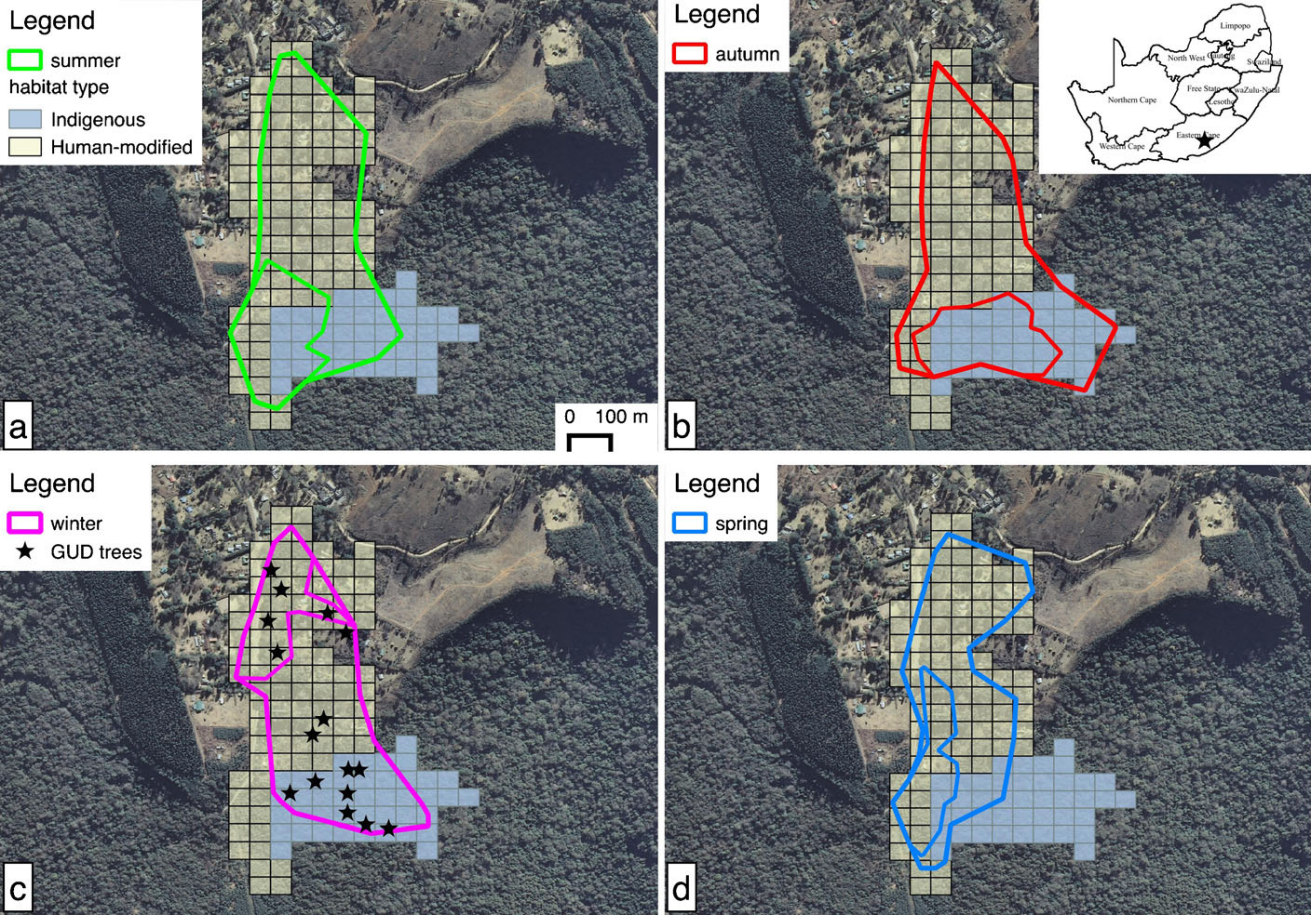
Polygons represent monkeys’ maximum and core ranges (100 % and 50 % isopleths) for each season with green **(a)** =summer, red **(b)** =autumn, pink **(c)** =winter, blue **(d)** =spring. Stars indicate locations of GUD patches that were established at random points generated inside 100 % of the monkeys’ winter range. (Note: the GUD experiment took place only in a subsequent winter.) The grid shows the total annual home range where off-white cells indicate human-modified habitat (including parts of Hogsback village) and light blue indicates indigenous forest

Hogsback lies in the Amathole Mountain range (32°35′S, 26°56′E) in the Eastern Cape province of South Africa (Fig. 1) at *ca.* 1200 m a.s.l. The village consists of large residential gardens planted primarily with exotic plant species including oak (especially *Quercus robur* and *Q. palustris*) and black wattle (*Acacia mearnsii*). The village is surrounded by indigenous, primarily southern mistbelt forest and commercial plantations of exotic pine (*Pinus* sp.). Mean annual rainfall is 1029 (±170 SD, *N* = 3 years) mm (Webster, *unpubl. data*) and temperatures fall from 29.6 (±2.2 SD) °C in summer to 5.7 (±1.2 SD) °C in winter, when it usually snows (SAWS 2011 *unpubl. data*).

Apart from a pair of resident crowned eagles (*Stephanoaetus coronatus*), risk from natural predators such as leopards (*Panthera pardus*) is low because of human-induced changes to natural habitat and hunting and trapping of predators in surrounding cattle and sheep farming areas. Anthropogenic risks to samango monkeys are high and include risk of injury and death by domestic dogs in residential properties, persecution by landowners when monkeys eat from orchards and even houses, and electrocution along powerlines used by monkeys to navigate the gardens’ discontinuous canopy. Conflict between human and nonhuman primates (chacma baboons [*Papio ursinus*] and samango monkeys) has escalated over recent years, with perceived increases in boldness, aggression, and population size of samango monkeys as well as growing overlap between samango monkey home ranges and residential properties (Wimberger *pers. obs.*; Wimberger and Bidner 2012). Although some attempts have been made to raise the awareness of people in Hogsback about samango monkey behavior and ways to coexist with them, e.g., by securing vegetable gardens (Wimberger and Bidner 2012), some residents have recently made calls to the provincial nature conservation agency for help with managing “the samango problem.”

### Study Groups

An estimated eight samango monkey groups inhabit Hogsback village and adjacent forests (Wimberger *unpubl. data*). We focused on one group (*ca*. 35 individuals), whose home range spanned both residential gardens in Hogsback village and intact indigenous state forest. This group had never before been exposed to any field experiments.

### Annual Ranging Patterns and Ground Use

We followed the study group for 35 d over 12 mo (February 1, 2011–January 31, 2012), for a total of 386.8 observation hours, split into summer (6 d during February 2011, December 2011, January 2012), autumn (9 d, March–May 2012), winter (11 d, June–August 2012), and spring (9 d, September–November 2012) for analyses. During full-day (dawn until dusk) follows, we used instantaneous scan sampling at intervals of 10 min to record the activity, diet (presented in Wimberger *et al. in prep*.), and the estimated height above ground of as many individuals as possible within a 5-min period. Estimated height above ground was later categorized as “ground” (0–2 m) or “tree” (>2 m) to compare with our GUD experiment (see later). We also recorded the group’s location every 30 min standing at the group center with a hand-held GPS (Dakota 20, Garmin Inc., USA).

We projected movement data (*N* = 230 in winter, 228 in spring, 161 in summer, 197 in autumn) in UTM Zone 35S, spheroid WGS 1984 before analyses. We used Fixed *k* Local Convex Hull (LoCoH, 2005. Wayne Getz lab. http://locoh.cnr.berkeley.edu/) to determine 100 % and 50 % (core) seasonal home ranges, because this method takes into account geomorphological boundaries such as roads (Getz *et al*. 2007). We used a *k* of 40, and duplicate points were displaced by one unit, i.e., in a random direction by 1 m, for analyses. We also determined the average mean daily distance moved by each group by calculating the distance between successive GPS positions using the Home Range Tools extension version 1.1 (Rodgers *et al*. 2007) for ESRI^®^ ArcMap^TM^ 9.3.1 (Esri 2008), which was then summed for each day. Where data points were missing (maximum of four data points), we calculated the distance from the last point recorded and the results thus show the minimum distance traveled each day. Using Hawth’s Analysis Tools 3.27 (Beyer 2004) extension for ESRI^®^ ArcMap^TM^ 9.3.1, we overlaid a grid on the GPS data points. A grid cell size of 50 × 50 m was chosen based on an estimate of mean group spread. For analysis of relative habitat use by each group, we labeled each cell as either “indigenous” or “human-modified” based on whether indigenous or exotic plants were dominant (>50 %) as determined through visual assessment based on satellite imagery and on the ground confirmation using resource abundance transects. We established these transects (100 m long with a width of 5 m on either side) throughout the home range of the group, and recorded the species, height, and diameter at breast height (DBH) of all trees with >5 cm DBH (Wimberger *et al. in prep*.).

### Experimental Food Patches in Forest and Gardens During Winter

We carried out the GUD experiment in winter 3 yr after behavioral and ranging data were collected, from May until July 2014. This was the food-scarce season (Wimberger *et al. in prep*.) when we would predict monkeys to take risks unless other options are available. We first generated 16 random points in QGIS (2.4.0. Chugiak, http://qgis.org, http://creativecommons.org/licenses/by-sa/3.0/) in the winter range of the study group (based on 100 % isopleth). We established eight experimental (GUD) food patches in exotic gardens and eight in indigenous forest (Fig. 1c, black asterisks on winter map). Food patches were established in a way consistent with previous GUD work on samango monkeys (*Cercopithecus albogularis schwarzi*) conducted in the Soutpansberg Mountains, Limpopo Province (Nowak *et al*. 2014). At each of the 16 locations, we suspended one plastic basin at each of the four heights, namely at the ground (0.1 m) to tree level at 2.5 m, 5 m, and 7.5 m, such that there were 64 experimental basins in total.

Before the experiment, we carried out 2 weeks of habituation, giving monkeys time to learn the location of the patches. In week 1, monkeys had access for 4 consecutive days to empty basins containing unshelled whole peanuts and orange quarters (used as extra incentive to draw monkeys in to the experimental area). In week 2, we increased the difficulty by restricting their access to the basins by weaving ropes along the top of the basins to slow foraging rate. We needed to influence the foraging rate so that monkeys would leave some food and we would have data on how much monkeys “gave up,” i.e., GUDs. During this week 2, we had 2 d when basins were filled with shelled whole peanuts and 1 L of sawdust and two ropes along the top and 2 days with halved peanuts in 2 L of sawdust with six ropes along the top. We then carried out 20 d of GUDs (4 d/week over 5 weeks) with 25 raw peanut halves mixed into 4 L of sawdust and a complex 12-cell grid of ropes along the top of the basin. GUD was the number of peanuts remaining at the end of each experimental day (16:00 h) and represented the extent to which patches were depleted. We analyze data from only these 20 experimental days.

### Analysis

We used nonparametric Kruskal–Wallis tests to examine seasonal differences in the time monkeys spent in gardens (fraction of total number of GPS points recorded during behavioral follows), and daily distance traveled. *Post hoc* tests were done on pairwise comparisons between seasons using the Tukey and Kramer (Nemenyi) test with Tukey–Dist approximation for independent samples data.

We fit a generalized linear mixed model (GLMM) with a logit link function and a binomial error distribution to the foraging data describing seasonal variation in ground use and a likelihood ratio test (LRT) used to test for seasonal differences. Though we retained the four basin heights in our analyses, we focused on two height categories: “ground” and “tree,” as our interest was to determine when arboreal monkeys would visit the risky ground vs. being safer on a tree, with 2 m representing a height where dogs and humans are unlikely to reach. Furthermore, a similar GUD experiment on a northern population of samango monkeys *Cercopithecus albogularis schwarzi* (Nowak *et al*. 2014) suggested that the biggest differences observed in GUDs were between experimental basins placed at ground vs. tree level.

We investigated the GUD data using GLMMs with a logit link function and a binomial error distribution. We considered basin height to be a covariate and location to be a fixed factor with two categories: gardens and forest. Our investigation of the data suggested that the role of day was best modeled as a random effect (Electronic Supplementary Materials), and we also included tree as a random effect. The GUD data were overdispersed with respect to the binomial distribution, so we accounted for this by including an additional random effect at the observation scale (Electronic Supplementary Materials). We used an LRT to test for an interaction between height and location. Finally, we used a GLMM (logit link function, binomial error distribution) and a LRT to compare rates of visitation between gardens and forest for our GUD experiment.

We performed all GLMM analyses in R 3.2.0 (R Core Team 2015) using the package lme4. When estimating uncertainty in our model predictions, we used bootstrapping to estimate 95 % confidence intervals (CIs). A full description of the analyses can be found in the Electronic Supplementary Materials.

## Ethical Note

Our research did not involve direct contact with monkeys during the behavioral follows or the GUD experiment. To limit potential pathogen transmission between researchers and monkeys, we used goggles and surgical masks to cover our faces when handling the food, and ensured we washed our hands before and after handling the food. Monkeys were “provisioned” only during this single and short experimental period, using the minimum number of peanuts needed to conduct the experiment, as there are possible negative implications of provisioning monkeys, including increased foraging in gardens and reduced fear of humans. The behavioral research (2010–2012) was approved by the National Zoological Gardens of South Africa’s Research and Ethics Committee and the University of Fort Hare, while the GUD research was approved by the Life Sciences Ethical Review Process Committee and Anthropology Department’s Ethics Subcommittee at Durham University and by the Interfaculty Animal Ethics Committee at the University of the Free State. Fieldwork was conducted with permission from the Department of Economic Development, Environmental Affairs and Tourism, and the Department of Agriculture, Forestry and Fisheries, Eastern Cape Province.

## Results

### Annual Ranging Patterns Across Forest and Gardens

Monkeys used residential gardens extensively (Fig. 1), but their use of gardens varied by season (Kruskal–Wallis *χ* ^2^ = 18.717, df = 3, *N* = 820 GPS points, *P* < 0.001), with gardens being used significantly more in spring than in autumn (*P* < 0.001). If only core ranges (50 % isopleths) are examined (Fig. 1), the seasonal differences in range overlap with human-modified habitat and indigenous forest can be seen more clearly [Kruskal–Wallis *χ* ^2^ = 253.21, df = 3, *N* = 396 GPS points, *P* < 0.001, with winter distinct from autumn (*P* < 0.001) and summer (*P* = 0.027) but not spring (*P* = 0.891) in the extent to which monkeys used gardens vs. forest]. During spring (Fig. 1d) and summer (Fig. 1a), monkeys’ core range included both human-modified habitat and indigenous forest, but in autumn (Fig. 1b) the group’s core range spanned indigenous habitat only, whereas in winter (Fig. 1c), the monkeys’ core range fell entirely inside gardens. The group’s core ranges were the smallest in spring (4.24 ha) and largest (6.37 ha) in summer and similarly sized in winter (4.70 ha) and autumn (4.94 ha).

We found no significant differences in mean daily path lengths across seasons (Kruskal–Wallis *χ* ^2^ = 5.492, df = 3, *N* = 33 days, *P* = 0.139) but the longest daily path length was in autumn (1360 ± 377 m SD), with the shortest in winter (1065 ± 234 m SD), compared with summer (1339 ± 189 m SD) and spring (1092 ± 144 m SD).

### Extent of Ground Use Across Seasons

**Fig. 2:**
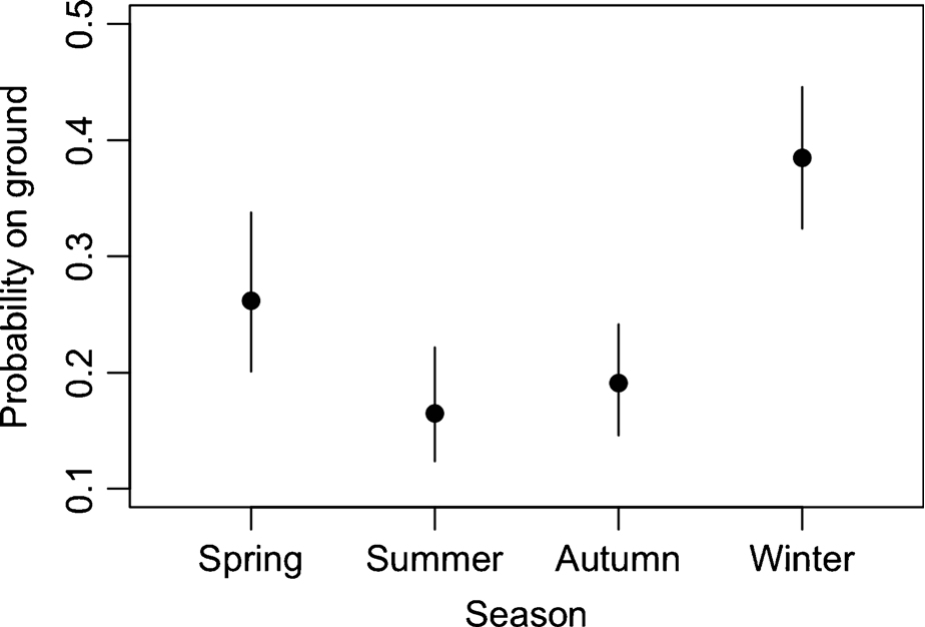
Mean (±95 % CI) proportion of records (*N* = 13,060 individual scans) collected during 35 days of group follows during which we observed monkeys on the ground, rather than in trees, across seasons

The extent to which monkeys used the ground differed across seasons (LRT, *G*_3_ = 21.20, *P* < 0.001; Fig. 2), with monkeys observed on the ground more frequently in winter, especially when compared with summer and autumn. Only in winter did monkeys spend more than a third of their time on the ground (Fig. 2).

### Relative Use of Food Patches in Forest vs. Gardens During Winter

**Fig. 3:**
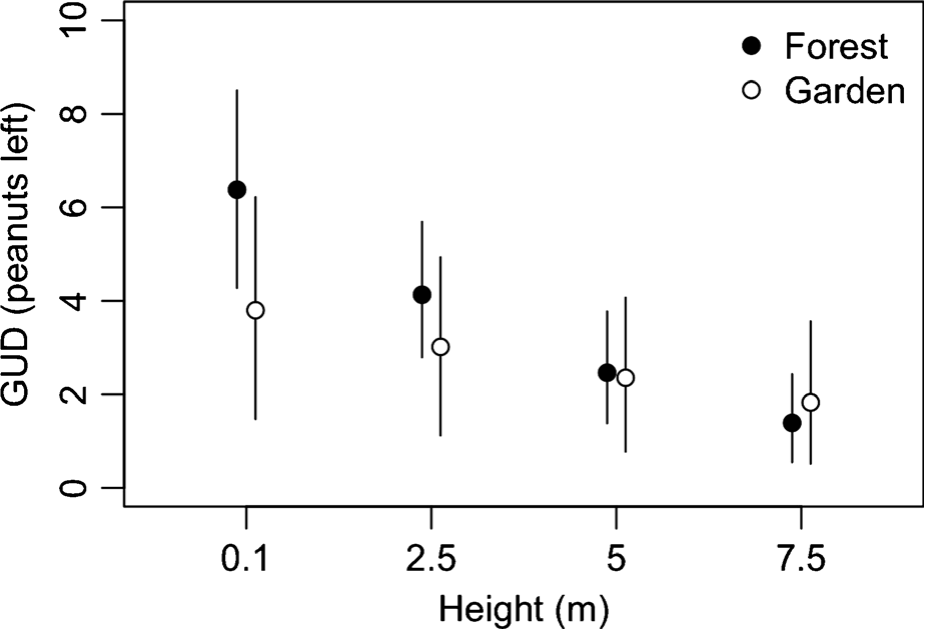
Mean (±95 % CI) GUD (peanuts left uneaten) by height and habitat

**Fig. 4:**
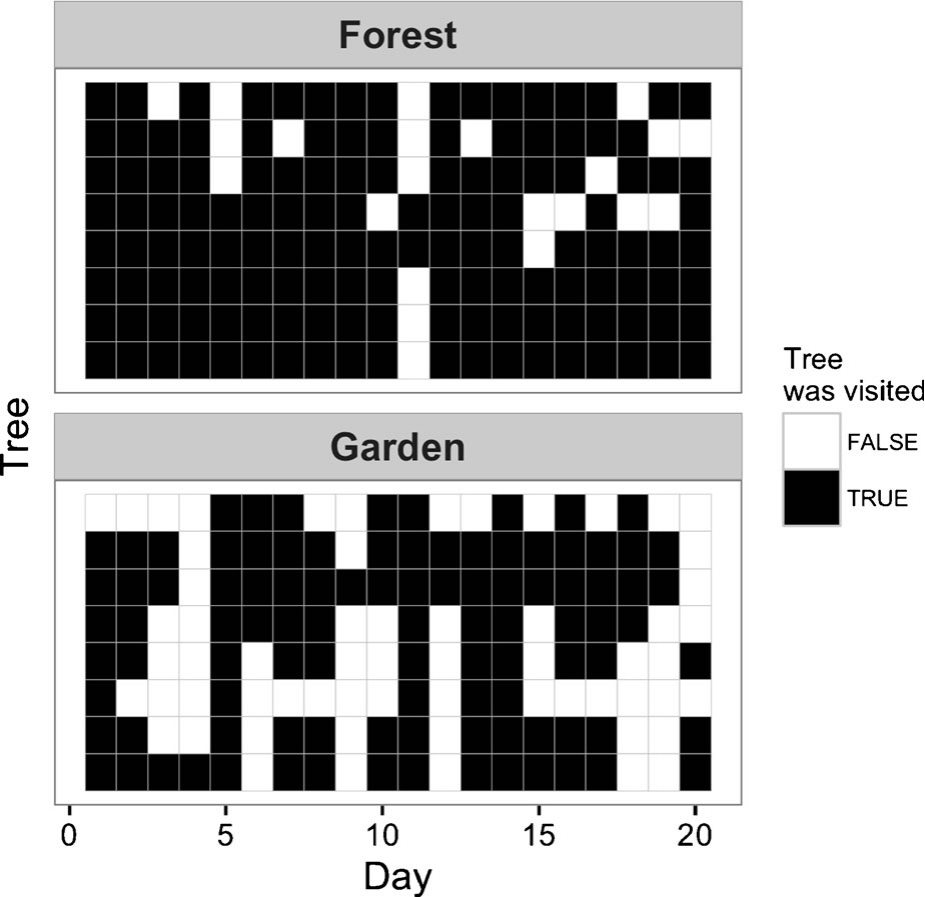
Monkeys’ patterns of visitation to GUD trees by habitat over 20 experimental days (data plotted by individual trees or GUD patches with 8 trees/patches per each habitat) showing that monkeys had higher visitation rates to experimental trees inside the forest than in gardens

We found an interaction between habitat (forest vs. gardens) and basin height (ground vs. tree) when predicting GUDs (LRT; *G*_1_ = 4.55, *P* = 0.033). GUDs were higher on the ground compared with the three tree levels in both habitats, and higher in forest habitat, especially for basins placed near the ground (Fig. 3). Despite GUDs being slightly lower in garden trees, we found evidence that trees in the forest were more often visited (LRT, *G*_1_ = 7.62, *P* = 0.006; Fig. 4), suggesting monkeys preferred to eat inside the forest than in the gardens.

## Discussion

Commensurate with our predictions, samango monkeys used gardens and the ground more extensively during winter, when forest food (indigenous fruit) availability is relatively lower (Wimberger *et al. in prep*.). Given equal foraging opportunities in the form of artificial foraging patches in both forest and garden habitats, and at positions on the ground and in trees, monkeys decreased their risk-taking behavior, changing their relative use of the matrix by foraging high in trees within indigenous forest. Higher visitation rates to forest patches suggested that monkeys perceived gardens and the ground to be riskier than indigenous forest and tree canopy level. However, this difference in perceived risk between habitats was not detectable when we compared the extent to which monkeys depleted peanuts (GUD) on actual visits to our experimental patches.

In the relatively food-rich autumn (Wimberger *et al. in prep*.), the monkeys’ ranges overlapped least with human-modified habitat, yet in the food-scarce winter, they spent most of their time in the village. The monkeys thus made a state-dependent decision, behaving in ways that reduced their risk of starvation while constrained by perceived risks (injury or harm) from humans and domestic dogs. Monkeys faced these risks by foraging on the ground and spending time in a human-dominated landscape, but they traded off energy intake (from relatively high quality exotic acorns, and later our experimental peanuts) against mortality risk (Lima *et al*. 1985). Seasonal changes in food availability are likely the primary drivers of monkeys’ risk-taking at this extreme southerly site.

As we reduced the risk of starvation in the winter with our GUD experiment (offering high value peanuts in both habitats), we observed a preference (higher visitation rates) by monkeys for safer forest patches in line with our predictions. Furthermore, given food at both ground and tree level, monkeys preferred to forage at less riskier heights above ground confirming findings from similar experiments at a site with high (natural) predatory density (Nowak *et al*. 2014). Our findings support the theoretical predictions of Sih (1980) that animals will make risk-averse decisions when they can. Our results also indicate that gardens are not inherently “preferred” or favored by monkeys, although exotic seeds are certainly attractive fallback foods (Wimberger *et al. in prep*.).

For monkeys to persist at this highly seasonal and human-modified site, they have learned to exploit the fallen exotic seeds in gardens during winter months when they are food limited in the forest. Gradually, they have become habituated to anthropogenic disturbance including tree canopy gaps and anthropogenic noise, e.g., radios and chainsaws. This habituation may help explain why monkeys depleted patches to the same extent in the gardens as in the forest once they had already decided to enter gardens.

A recent study on the effects of human noise on ungulates using roadside surveys and observations of elk and pronghorn (*Antilocapra americana*) along a road corridor in Grand Teton National Park, Wyoming, USA, found that elk were less vigilant and less likely to flee and exhibit defensive behavior with increasing levels of vehicle traffic (Brown *et al*. 2012). They did however respond to visible moving threats such as pedestrians and passing motorcycles while continuing to ignore the “background noise.” This suggests that noise and human activity were not necessarily associated with increased predation risk nor could heightened responsiveness to frequent human stimuli be maintained (Brown *et al*. 2012). Likewise, monkeys distinguish between different types, levels, and frequencies of anthropogenic risk and respond appropriately (Nowak *et al*. 2016). Because risk tends to increase as animals move into new areas, monkeys may opt to remain in familiar locations to reduce perceived risk; and, as their experience in an area, e.g., gardens, grows, they may also increase their willingness to exploit patches to higher extents (rather than move to new, potentially riskier, locations). Monkeys did have slightly lower GUDs in the gardens than in the forest, indicating that once they had taken the risk to enter gardens, they ate as much as possible. The relatively higher depletion of garden patches could also be explained by monkeys not moving as much or as far in the gardens given the clumped nature of exotic foods (Wimberger *et al. in prep*.), as our ranging data show.

Human presence is not always disadvantageous to prey species given that it may come to be associated with lower natural predation risk (Berger 2007; Nowak *et al*. 2014) as people displace terrestrial predators such as leopards (Isbell and Young 1993). In Hogsback gardens, where dogs pose a real risk, the presence of property owners who like having monkeys in their gardens may confer safety if these people discourage dogs from chasing monkeys. Two monkeys (including one monkey from this group) have been attacked by dogs, while other instances of dogs killing monkeys have been reported by Hogsback residents (Wimberger *unpubl. data*). Accordingly, monkeys may perceive small-scale differences in spatial risk and show preference for certain gardens. People also more readily chase baboons—a probable competitor of samango monkeys at this site—as baboons are seen as dangerous to people and domestic animals more so than the samango monkeys, and samangos may therefore perceive gardens as a “baboon-free” zone. During our GUDs experiments, we observed samango monkeys moving off in complete silence on detecting incoming baboons (while in the forest), suggesting that monkeys were willing to abandon patches of peanuts to avoid baboons.

That monkeys do not generally avoid gardens does not mean that they are not negatively affected, i.e., stressed, or deterred by human presence and disturbance, or that they do not need protection (Gill *et al*. 2001). Increased commensalism at this site could ultimately adversely affect human–monkey relationships, the physical health of monkeys, e.g., dentition (Tordiffe *et al. unpubl. data*), and samango monkey population size in Hogsback. Over the past 5 or so years, human–monkey conflict has increased as samango monkeys have ventured more frequently and extensively into residential properties (Wimberger *pers. obs*.; Wimberger and Bidner 2012). The removal of raked piles of fallen acorns (which represent highly concentrated food patches) and exotic seeds from gardens during winter, as well as covering up rubbish and vegetable gardens, are potential mitigation strategies that could help deter monkeys from gardens (see Wimberger and Bidner 2012 for further recommendations). Long-term solutions will require the gradual phasing out of exotic species that people have planted inside gardens (Wimberger *et al*., *this issue*). A concurrent study of monkeys’ neophilia (Mathibane 2014) suggested that this same group was more interested in anthropogenic objects, e.g., plastic toys, in the gardens than in the forest. As a consequence, possible intervention strategies aimed at deterring monkeys from gardens may be complicated further by this differential response of monkeys to people and their objects in gardens, which suggests a reduced fear or even elevated neophilia in this relatively novel and fluctuating habitat.

We are optimistic that given improved human understanding of monkeys’ habitat choices in Hogsback, and some relatively minor changes in people’s habits and maintenance of properties, monkey–human coexistence can be sustained at this highly unusual site where samango monkeys manage risks in a human-modified landscape and endure the pronounced winters at what is the southern limit of their biogeographic range.

## Electronic supplementary material

Below is the link to the electronic supplementary material.

ESM 1 (DOCX 293 kb)
