# Supplementary material for: Samango Monkeys (Cercopithecus albogularis labiatus) Manage Risk in a Highly Seasonal, Human-Modified Landscape in Amathole Mountains, South Africa
Source: Int J Primatol. 2016 Aug 19;38(2):194–206. doi: 10.1007/s10764-016-9913-1 (PMC5422488; doi:10.1007/s10764-016-9913-1)
Supplement: Supplementary file 1 — (DOCX 293 kb) [file 10764_2016_9913_MOESM1_ESM.docx]

# Electronic Supplementary Material

**Samango Monkeys (*Cercopithecus albogularis labiatus*) Manage Risk in a Highly Seasonal, Human-Modified Landscape in Amathole Mountains, South Africa**

**Katarzyna Nowak ∙ Kirsten Wimberger ∙ Shane A. Richards ∙ Russell A. Hill ∙ Aliza le Roux^b^**

# Introduction

This document provides the analyses of the data presented in the article using the statistical programming language R. Three statistical analyses are presented here:

1. Predict differences in ground vs. tree visits across seasons.
2. Predict effects of basin height and location on giving-up densities (GUDs).
3. Predict effects of location and day on tree visitation.

All three analyses involve use of generalized linear mixed-effects models (GLMMs) with a binomial error distribution and a logit link function.

## Data

Read in the relevant data sets and store them in data frames for analyses. Some cleaning of the data is also required, e.g., setting variable types.

rm(list = ls()) # clear memory

library(dplyr)
library(ggplot2)
library(lme4)

setwd("~/Documents/Projects/Primates/Seasonal Risk/")

# read in the ground/tree observational data for analysis 1
df.Season <- read.csv(file = "GroundR.csv",header = TRUE)
df.Season$Season <- factor(df.Season$Season,
 levels(df.Season$Season)[c(2,3,1,4)]) # reorder Season levels
glimpse(df.Season)

## Observations: 35
## Variables: 5
## $ Obs (int) 1, 2, 3, 4, 5, 6, 7, 8, 9, 10, 11, 12, 13, 14, 15, 16, ...
## $ Tree (int) 232, 286, 273, 303, 249, 243, 230, 282, 201, 180, 334, ...
## $ Ground (int) 78, 39, 88, 25, 29, 78, 47, 77, 81, 158, 247, 204, 227,...
## $ Date (fctr) 3-Mar, 4-Mar, 31-Mar, 8-Apr, 18-Apr, 22-Apr, 28-Apr, 5...
## $ Season (fctr) Autumn, Autumn, Autumn, Autumn, Autumn, Autumn, Autumn...

# read in data for analyzes 2 and 3
df.GUD <- read.csv(file = "VillageGUDs_visitsonly.csv",header = TRUE)
# keep data relevant to the analysis
df.GUD <- df.GUD %>% select(Tree,Location,BasinHeight,ExpDay,PeanutsLeft,Ht)
df.GUD$BasinHeightF <- factor(df.GUD$BasinHeight)

# generate visit data frame for binomial regression
N <- 8*2*20 # trees * locations * Days
Visited.Tree <- matrix(data = FALSE, ncol = 16, nrow = 20)
GUD <- array(data = 25, dim = c(20,16,4),
 dimnames = c("ExpDay", "Tree", "BasinHeight"))

# add visits
for (i in 1:dim(df.GUD)[1]) {
 tree.id <- df.GUD$Tree[i]
 day <- df.GUD$ExpDay[i]
 ht <- as.integer(df.GUD$BasinHeightF[i])
 Visited.Tree[day,tree.id] <- TRUE # at least one visit made to tree on day
 GUD[day, tree.id, ht] <- df.GUD$PeanutsLeft[i]
}

Visited.Basin <- (GUD < 25) # visited if some peanuts taken

df.Visit <- data.frame(Day = 1:20, Visited.Tree) # binom regression (test 3)
names(df.Visit)[2:17] <- 1:16
df.Visit <- tidyr::gather(data = df.Visit, key = Tree, value = Visited, 2:17)
df.Visit$Tree <- as.integer(df.Visit$Tree)
df.Visit <- df.Visit %>% mutate(Location = ifelse(Tree <= 8, "G", "F"))
df.Visit$Location <- factor(df.Visit$Location)
df.Visit$Tree <- factor(df.Visit$Tree)

df.GUD$Tree <- factor(df.GUD$Tree) # make sure tree is a factor
df.GUD$PeanutsInitial <- rep(25, dim(df.GUD)[1]) # add init number of peanuts
df.GUD$Obs <- 1:dim(df.GUD)[1] # dummy factor to account for overdispersion

glimpse(df.GUD)

## Observations: 899
## Variables: 9
## $ Tree (fctr) 1, 3, 4, 5, 6, 7, 9, 10, 11, 12, 16, 5, 10, 12...
## $ Location (fctr) G, G, G, G, G, G, F, F, F, F, F, G, F, F, F, F...
## $ BasinHeight (dbl) 0.1, 0.1, 0.1, 0.1, 0.1, 0.1, 0.1, 0.1, 0.1, 0....
## $ ExpDay (int) 1, 1, 1, 1, 1, 1, 1, 1, 1, 1, 1, 2, 2, 2, 2, 2,...
## $ PeanutsLeft (int) 21, 25, 21, 1, 1, 1, 3, 10, 4, 0, 2, 17, 0, 9, ...
## $ Ht (fctr) Ground, Ground, Ground, Ground, Ground, Ground...
## $ BasinHeightF (fctr) 0.1, 0.1, 0.1, 0.1, 0.1, 0.1, 0.1, 0.1, 0.1, 0...
## $ PeanutsInitial (dbl) 25, 25, 25, 25, 25, 25, 25, 25, 25, 25, 25, 25,...
## $ Obs (int) 1, 2, 3, 4, 5, 6, 7, 8, 9, 10, 11, 12, 13, 14, ...

glimpse(df.Visit)

## Observations: 320
## Variables: 4
## $ Day (int) 1, 2, 3, 4, 5, 6, 7, 8, 9, 10, 11, 12, 13, 14, 15, 16...
## $ Tree (fctr) 1, 1, 1, 1, 1, 1, 1, 1, 1, 1, 1, 1, 1, 1, 1, 1, 1, 1...
## $ Visited (lgl) TRUE, TRUE, TRUE, TRUE, TRUE, FALSE, TRUE, TRUE, FALS...
## $ Location (fctr) G, G, G, G, G, G, G, G, G, G, G, G, G, G, G, G, G, G...

# Analysis 1: Seasonal Patterns GLMM

Season is a fixed factor and overdispersion is accounted for by including a random factor that is influential at the observation level. We fit the GLMM and use a likelihood ratio test (LRT) to look for evidence of a main effect.

model <- glmer(cbind(Ground, Tree) ~ Season + (1 | Obs),
 family=binomial(link=logit), data = df.Season)

summary(model)

## Generalized linear mixed model fit by maximum likelihood (Laplace
## Approximation) [glmerMod]
## Family: binomial ( logit )
## Formula: cbind(Ground, Tree) ~ Season + (1 | Obs)
## Data: df.Season
##
## AIC BIC logLik deviance df.resid
## 352.4 360.2 -171.2 342.4 30
##
## Scaled residuals:
## Min 1Q Median 3Q Max
## -0.65633 -0.23557 0.01357 0.17643 0.41552
##
## Random effects:
## Groups Name Variance Std.Dev.
## Obs (Intercept) 0.2502 0.5002
## Number of obs: 35, groups: Obs, 35
##
## Fixed effects:
## Estimate Std. Error z value Pr(>|z|)
## (Intercept) -1.0974 0.1720 -6.381 1.76e-10 ***
## SeasonSummer -0.6122 0.2740 -2.235 0.02544 *
## SeasonAutumn -0.4253 0.2449 -1.737 0.08243 .
## SeasonWinter 0.5957 0.2309 2.580 0.00988 **
## ---
## Signif. codes: 0 '***' 0.001 '**' 0.01 '*' 0.05 '.' 0.1 ' ' 1
##
## Correlation of Fixed Effects:
## (Intr) SsnSmm SsnAtm
## SeasonSummr -0.628
## SeasonAutmn -0.702 0.441
## SeasonWintr -0.745 0.468 0.523

drop1(model, test = "Chisq") # LRT test for a season effect

## Single term deletions
##
## Model:
## cbind(Ground, Tree) ~ Season + (1 | Obs)
## Df AIC LRT Pr(Chi)
## <none> 352.39
## Season 3 367.59 21.201 9.564e-05 ***
## ---
## Signif. codes: 0 '***' 0.001 '**' 0.01 '*' 0.05 '.' 0.1 ' ' 1

There is evidence of seasonal differences.

## Bootstrapping of Seasonal data

Calculate the predicted mean proportional use and the 95% confidence interval using bootstrapping.

# generate bootstrapped data sets and fit GLMMM to each
BOOTSTRAP.SAMPLES <- 200 # number of bootstraps
REPS <- 10000 # number of random effect replicates used to calculate mean
# declare space for parameter estimates across bootstrap samples
beta.Sp <- rep(0, BOOTSTRAP.SAMPLES)
beta.Su <- rep(0, BOOTSTRAP.SAMPLES)
beta.Au <- rep(0, BOOTSTRAP.SAMPLES)
beta.Wi <- rep(0, BOOTSTRAP.SAMPLES)
sigma.r <- rep(0, BOOTSTRAP.SAMPLES)
p.Sp <- rep(0, BOOTSTRAP.SAMPLES)
p.Su <- rep(0, BOOTSTRAP.SAMPLES)
p.Au <- rep(0, BOOTSTRAP.SAMPLES)
p.Wi <- rep(0, BOOTSTRAP.SAMPLES)
# prepare dataframe to store bootstrapped predictions
Obs <- 1:35
Tree <- rep(0,35)
Ground <- rep(0,35)
Season <- c(rep("Autumn",9),rep("Winter",11),rep("Spring",9),rep("Summer",6))
df.bs <- data.frame(Obs, Tree, Ground, Season)

for (bs in 1:BOOTSTRAP.SAMPLES) {
 # create a boostrapped sample for fitting
 Obs.Au <- sample(1:9,9,replace=TRUE)
 Obs.Wi <- sample(10:20,11,replace=TRUE)
 Obs.Sp <- sample(21:29,9,replace=TRUE)
 Obs.Su <- sample(30:35,6,replace=TRUE)
 Obs.r <- c(Obs.Au, Obs.Wi, Obs.Sp, Obs.Su)
 for (i in 1:35) { # observation
 df.bs$Tree[i ] <- df.Season$Tree[Obs.r[i]]
 df.bs$Ground[i] <- df.Season$Ground[Obs.r[i]]
 }
 # fit the same GLMM to the bootstrapped data set
 model <- glmer(cbind(Ground, Tree) ~ Season + (1 | Obs),
 family=binomial(link=logit), data = df.bs)

 # Store the best-fit parameters
 beta.Au[bs] <- fixef(model)[1] # F (intercept)
 beta.Sp[bs] <- fixef(model)[2] # add to get G (intercept)
 beta.Su[bs] <- fixef(model)[3] # H (slope)
 beta.Wi[bs] <- fixef(model)[4] # add to get F (slope)
 sigma.r[bs] <- as.data.frame(VarCorr(model))$sdcor[1] # random effect sd

 # generate REPS observations based on GLMM fit (include the random effect)
 rv <- rnorm(REPS, mean = 0, sd = sigma.r[bs])
 linear.Au <- beta.Au[bs] + rv
 rv <- rnorm(REPS, mean = 0, sd = sigma.r[bs])
 linear.Sp <- beta.Au[bs] + beta.Sp[bs] + rv
 rv <- rnorm(REPS, mean = 0, sd = sigma.r[bs])
 linear.Su <- beta.Au[bs] + beta.Su[bs] + rv
 rv <- rnorm(REPS, mean = 0, sd = sigma.r[bs])
 linear.Wi <- beta.Au[bs] + beta.Wi[bs] + rv
 # calculate the mean probability from the REPS replicates and store
 p.Sp[bs] <- mean(exp(linear.Sp)/(1+exp(linear.Sp)))
 p.Su[bs] <- mean(exp(linear.Su)/(1+exp(linear.Su)))
 p.Au[bs] <- mean(exp(linear.Au)/(1+exp(linear.Au)))
 p.Wi[bs] <- mean(exp(linear.Wi)/(1+exp(linear.Wi)))
}

# create a dataframe of the bootstrapped means for manipulation and plotting
Season <- c(rep("Spring",BOOTSTRAP.SAMPLES), rep("Summer",BOOTSTRAP.SAMPLES),
 rep("Autumn",BOOTSTRAP.SAMPLES),rep("Winter",BOOTSTRAP.SAMPLES))
Probability <- c(p.Sp, p.Su, p.Au, p.Wi)
df.plot <- data.frame(Season, Probability)
df.plot$Season <- factor(df.plot$Season,
 levels(df.plot$Season)[c(2,3,1,4)])# reorder Season levels
# calculate the mean and 95% CI from the bootstrapped means
df.summary <- df.plot %>%
 group_by(Season) %>%
 summarise(low95 = quantile(Probability, 0.025),
 mean = mean(Probability),
 high95 = quantile(Probability, 0.975))

# store bootstrapped results for later (creating figure 2 for the manuscript)
write.csv(df.summary, file = "bootstrapSeason.csv", row.names = FALSE)

## Create Season Figure for paper (Fig. 2)

# read in the bootstrapped results
df.summary <- read.csv(file = "bootstrapSeason.csv", header = TRUE)

ggplot(df.summary, aes(x = Season, y = mean)) +
 geom_point() +
 geom_errorbar(aes(ymin=low95, ymax=high95), width=.2) +
 theme_bw()


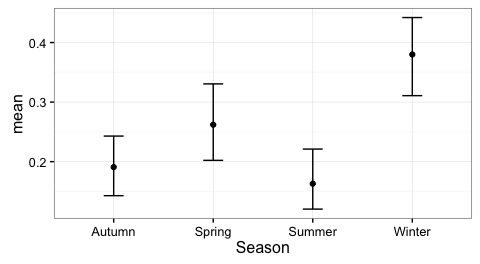


Trees are more frequently used relative to ground, and tree use in spring and winter is lower relative to that in summer and autumn.

# create a jpeg for publication
jpeg("bootstrapSeason.jpeg", height = 1000, width = 1400, res = 300)
par(mar=c(4.1, 4.1, 1.1, 1.1))
dx <- 0.05 # amount to offset garden versus forest estimates
plot(x = 1:4, y = df.summary$mean, pch = 21,
 bg = "black", ylim = c(0.1,0.5), xlim = c(0.5,4.5),
 xlab = "", ylab = "", xaxt = "n")
segments(x0 = 1:4, y0 = df.summary$low95, x1 = 1:4, y1 = df.summary$high95)
axis(1, at=1:4, labels=c("Spring", "Summer","Autumn","Winter"))
mtext(text = "Season", side = 1, line = 2.5)
mtext(text = "Probability on ground", side = 2, line = 2.5)
dev.off()

## quartz_off_screen
## 2

# Analysis 2: GUD GLMM

First, plot the data to determine the most appropriate model structure. How should day be treated: a covariate or a random effect?

df.plot <- df.GUD %>% group_by(Location, BasinHeight, ExpDay) %>%
 summarise(n = n(), meanGUD = mean(PeanutsLeft))

ggplot(df.plot, aes(x = ExpDay, y = meanGUD, color = Location)) +
 geom_point() + geom_line() +
 facet_wrap( ~ BasinHeight) +
 xlab("Day") + ylab("GUD (peanuts left)") +
 theme_bw()


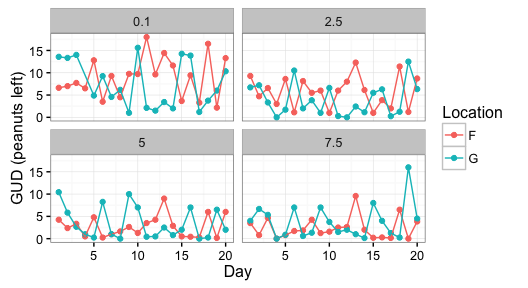


df.plot <- df.GUD %>% group_by(Location, Tree, ExpDay) %>%
 summarise(n = n(), meanGUD = mean(PeanutsLeft))

ggplot(df.plot, aes(x = ExpDay, y = meanGUD, color = Tree)) +
 geom_point() + geom_line() +
 facet_wrap( ~ Location) +
 guides(col = guide_legend(ncol = 2)) +
 xlab("Day") + ylab("GUD (peanuts left)") +
 theme_bw()


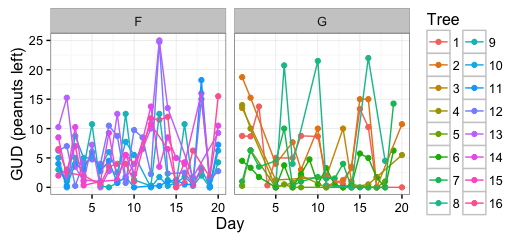


There is no clear evidence of a consistent temporal trend in GUDs; however, there is some evidence that GUDs correlate across trees on a given day. Thus, day will be included as a random effect rather than a covariate.

df.plot <- df.GUD %>%
 group_by(Location, BasinHeight) %>%
 summarise(n = n(), mean.GUD = mean(PeanutsLeft)) # observed mean GUD

ggplot(df.plot, aes(x = BasinHeight, y = mean.GUD, color = Location)) +
 ylim(0,10) + xlab("Basin height (m)") + ylab("GUD (peanuts)") +
 geom_point() + # observed
 theme_bw()


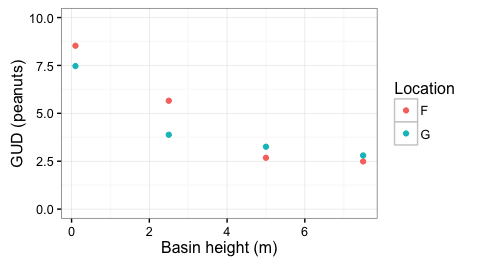


GUDs were similar but slightly higher in the forest relative to the garden, especially at lower heights.

Fit the GLMM and look for evidence of an interaction using an LRT.

model <- glmer(cbind(PeanutsLeft, PeanutsInitial-PeanutsLeft) ~
 Location + BasinHeight + Location:BasinHeight +
 (1 | Tree) + (1 | Obs) + (1 | ExpDay),
 family=binomial(link=logit), data = df.GUD)

summary(model)

## Generalized linear mixed model fit by maximum likelihood (Laplace
## Approximation) [glmerMod]
## Family: binomial ( logit )
## Formula: cbind(PeanutsLeft, PeanutsInitial - PeanutsLeft) ~ Location +
## BasinHeight + Location:BasinHeight + (1 | Tree) + (1 | Obs) +
## (1 | ExpDay)
## Data: df.GUD
##
## AIC BIC logLik deviance df.resid
## 4064.2 4097.8 -2025.1 4050.2 892
##
## Scaled residuals:
## Min 1Q Median 3Q Max
## -0.82076 -0.39999 -0.08055 0.09529 0.95392
##
## Random effects:
## Groups Name Variance Std.Dev.
## Obs (Intercept) 6.7552 2.5991
## ExpDay (Intercept) 0.7049 0.8396
## Tree (Intercept) 1.5762 1.2555
## Number of obs: 899, groups: Obs, 899; ExpDay, 20; Tree, 16
##
## Fixed effects:
## Estimate Std. Error z value Pr(>|z|)
## (Intercept) -1.19612 0.52484 -2.279 0.0227 *
## LocationG -0.84444 0.71659 -1.178 0.2386
## BasinHeight -0.48502 0.04899 -9.900 <2e-16 ***
## LocationG:BasinHeight 0.16248 0.07616 2.134 0.0329 *
## ---
## Signif. codes: 0 '***' 0.001 '**' 0.01 '*' 0.05 '.' 0.1 ' ' 1
##
## Correlation of Fixed Effects:
## (Intr) LoctnG BsnHgh
## LocationG -0.639
## BasinHeight -0.292 0.225
## LctnG:BsnHg 0.190 -0.365 -0.621

drop1(model, test = "Chisq") # LRT test for an interaction effect

## Single term deletions
##
## Model:
## cbind(PeanutsLeft, PeanutsInitial - PeanutsLeft) ~ Location +
## BasinHeight + Location:BasinHeight + (1 | Tree) + (1 | Obs) +
## (1 | ExpDay)
## Df AIC LRT Pr(Chi)
## <none> 4064.2
## Location:BasinHeight 1 4066.8 4.5485 0.03295 *
## ---
## Signif. codes: 0 '***' 0.001 '**' 0.01 '*' 0.05 '.' 0.1 ' ' 1

There is some evidence of an interaction between Location and BasinHeight. This result implies that both Location and BasinHeight are statistically significant.

## Bootstrapping of GUD Data

Now calculate mean GUDs based on the regression parameter estimates. The logit link function means that the expected values for GUDs cannot be calculated from the beta-parameters alone; these parameters determine the mode, not the mean. The mean is dependent on the variances of the random effects. A customized bootstrapping approach is needed to calculate the mean.

# generate bootstrapped data sets and fit GLMMM to each
BOOTSTRAP.SAMPLES <- 200 # number of bootstraps
TREES <- 200 # tree replicates for estimating bias due to random effects
DAYS <- 100 # day reps per tree for estimating bias due to random effects
hts <- c(0.1,2.5,5.0,7.5) # basin heights
# declare space for parameter estimates across bootstrap samples
beta.0 <- rep(0, BOOTSTRAP.SAMPLES)
beta.H <- rep(0, BOOTSTRAP.SAMPLES)
beta.L <- rep(0, BOOTSTRAP.SAMPLES)
beta.LH <- rep(0, BOOTSTRAP.SAMPLES)
sigma.o <- rep(0, BOOTSTRAP.SAMPLES) # each observations
sigma.t <- rep(0, BOOTSTRAP.SAMPLES) # tree
sigma.d <- rep(0, BOOTSTRAP.SAMPLES) # day
# needed for mean estimates per bootstrap sample
bs.y <- matrix(data = 0.0, nrow = 2, ncol = 4)
bs.GUDs <- matrix(data = 0.0, nrow = 2, ncol = 4)
bs.mean <- array(data = 0.0, dim = c(BOOTSTRAP.SAMPLES,2,4))

# prepare dataframe to store bootstrapped predictions
bs.GUD <- rep(0, 16*4*20)
Tree <- sort(rep(1:16,4*20))
Location <- c(rep("G",8*4*20), rep("F",8*4*20))
BasinHeight <- rep(sort(rep(hts,20)),16)
ExpDay <- rep(1:20,16*4)
Obs <- 1:(16*4*20) # used for fitting random effect (overdispersion)
df.bs <- data.frame(Tree,Location,BasinHeight,ExpDay,PeanutsLeft = bs.GUD,
 PeanutsInitial = 25, Obs)

for (bs in 1:BOOTSTRAP.SAMPLES) {
 Trees.G <- sample(1:8,8,replace=TRUE)
 Trees.F <- sample(9:16,8,replace=TRUE)
 Trees <- c(Trees.G, Trees.F)
 Days <- sample(1:20,20,replace=TRUE)
 i <- 0
 for (tr in 1:16) { # tree
 for (ht in 1:4) { # basin height
 for (da in 1:20) { # day
 i <- i + 1
 df.bs$PeanutsLeft[i] <- GUD[Days[da],Trees[tr],ht]
 }
 }
 }
 # create data frame to fit
 df.bs.trimmed <- df.bs[df.bs$PeanutsLeft < 25, ] # fit to visited basins
 df.bs.trimmed$Tree <- factor(df.bs.trimmed$Tree) # Tree is a factor
 df.bs.trimmed$Location <- factor(df.bs.trimmed$Location) # a factor
 # fit the GLMM
 model <- glmer(cbind(PeanutsLeft, PeanutsInitial-PeanutsLeft) ~
 Location + BasinHeight + Location:BasinHeight +
 (1 | Tree) + (1 | Obs) + (1 | ExpDay),
 family=binomial(link=logit), data = df.bs.trimmed)
 # Store the best-fit parameters
 beta.0[bs] <- fixef(model)[1] # F (intercept)
 beta.L[bs] <- fixef(model)[2] # add to get G (intercept)
 beta.H[bs] <- fixef(model)[3] # H (slope)
 beta.LH[bs] <- fixef(model)[4] # add to get F (slope)
 sigma.o[bs] <- as.data.frame(VarCorr(model))$sdcor[1] # obs random eff sd
 sigma.d[bs] <- as.data.frame(VarCorr(model))$sdcor[2] # day random eff sd
 sigma.t[bs] <- as.data.frame(VarCorr(model))$sdcor[3] # tree random eff sd

 bs.GUDs[ , ] <- 0.0
 # estimate mean GUD for the bootsrap estimates
 for (tr in 1:TREES) {
 zt <- rnorm(1, mean = 0, sd = sigma.t[bs]) # tree random variable
 for (da in 1:DAYS) {
 zd <- rnorm(1, mean = 0, sd = sigma.d[bs]) # day random variable
 zo <- rnorm(4, mean = 0, sd = sigma.o[bs]) # overdispersion
 bs.y[1, ] <- beta.0[bs] + hts*beta.H[bs] +
 zo + zt + zd # forest (linear)
 zo <- rnorm(4, mean = 0, sd = sigma.o[bs]) # overdispersion
 bs.y[2, ] <- (beta.0[bs]+beta.L[bs]) + hts*(beta.H[bs]+beta.LH[bs]) +
 zo + zt + zd # garden (linear)
 bs.GUDs <- bs.GUDs + 25 * exp(bs.y) / (1.0 + exp(bs.y)) # cumulative
 }
 }
 bs.mean[bs, , ] <- bs.GUDs / (TREES*DAYS) # mean GUDs
}

## Warning in checkConv(attr(opt, "derivs"), opt$par, ctrl = control$checkConv, : Model is nearly unidentifiable: very large eigenvalue
## - Rescale variables?

# calculate the distribution of estimated mean GUDs
BasinHeight <- rep(c(hts,hts),BOOTSTRAP.SAMPLES)
mean.GUD <- NULL
Location <- NULL

# store the estimated means in a data frame
for (bs in 1:BOOTSTRAP.SAMPLES) {
 mean.GUD <- c(mean.GUD,bs.mean[bs,1, ],bs.mean[bs,2, ])
 Location <- c(Location, c("F","F","F","F","G","G","G","G"))
}
df.plot.error <- data.frame(Location, BasinHeight, mean.GUD)
df.plot$Location <- factor(df.plot$Location) # a factor for plotting

# plot the bootstrapped distibution of means
ggplot(data = df.plot.error,
 aes(x=factor(BasinHeight), y=mean.GUD, color = Location)) +
 ylim(0,12) +
 xlab("Basin height (m)") + ylab("GUD (peanuts remaining)") +
 geom_boxplot() +
 theme_bw()

## Warning: Removed 1 rows containing non-finite values (stat_boxplot).


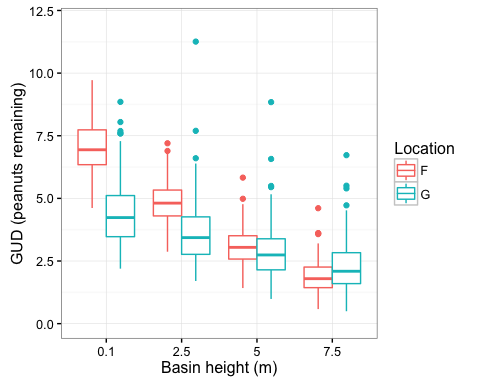


# create summary of bootstrapped means (this will be plotted in the paper)
df.summary <- df.plot.error %>%
 group_by(BasinHeight, Location) %>%
 summarise(low95 = quantile(mean.GUD, 0.025),
 mean = mean(mean.GUD),
 high95 = quantile(mean.GUD, 0.975))
# store the bootstrapped results for later
write.csv(df.summary, file = "bootstrapGUD.csv", row.names = FALSE)

# read in the bootstrapped results
df.summary <- read.csv(file = "bootstrapGUD.csv", header = TRUE)

ggplot(df.summary, aes(x=BasinHeight, y=mean, color = Location)) +
 geom_point() + geom_line() +
 geom_errorbar(aes(ymin=low95, ymax=high95), width=.2) +
 xlab("Basin height (m)") + ylab("Predicted GUD (peanuts left)") +
 theme_bw()


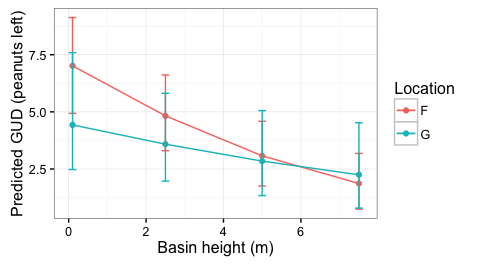


The model predicts high uncertainty in mean GUDs; however, the height effect is clear.

## Create GUD Figure for Paper (Fig. 3)

# create a jpeg for publication
jpeg("bootstrapGUD.jpeg", height = 1000, width = 1400, res = 300)
par(mar=c(4.1, 4.1, 1.1, 1.1))
dx <- 0.05 # amount to offset garden versus forest estimates
plot(x = 1:4-dx, y = filter(df.summary, Location == "F")$mean, pch = 21,
 bg = "black", ylim = c(0,10), xlim = c(0.5,4.5),
 xlab = "", ylab = "", xaxt = "n")
segments(x0 = 1:4-dx, y0 = filter(df.summary, Location == "F")$low95,
 x1 = 1:4-dx, y1 = filter(df.summary, Location == "F")$high95)
segments(x0 = 1:4+dx, y0 = filter(df.summary, Location == "G")$low95,
 x1 = 1:4+dx, y1 = filter(df.summary, Location == "G")$high95)
points(x = 1:4+dx, y = filter(df.summary, Location == "G")$mean, pch = 21,
 bg = "white")
axis(1, at=1:4, labels=c("0.1", "2.5","5","7.5"))
mtext(text = "Height (m)", side = 1, line = 2.5)
mtext(text = "GUD (peanuts left)", side = 2, line = 2.5)
legend("topright", legend = c("Forest", "Garden"), bty = "n", pch = 21,
 pt.bg = c("black", "white"))
dev.off()

## quartz_off_screen
## 2

# Analysis 3: Tree Visitation GLMM

First, plot the data to see how visitation varies both between trees and between days.

df.Visit <- df.Visit %>%
 mutate(Tree.plot = ifelse(as.integer(Tree) <= 8,Tree,as.integer(Tree)-8))
df.Visit <- df.Visit %>%
 mutate(Location.plot = ifelse(Location == "F", "Forest", "Garden"))

ggplot(df.Visit, aes(x = Day, y = Tree.plot, fill = Visited)) +
 geom_tile(color = "grey") +
 scale_fill_manual(values=c("white", "black")) +
 facet_wrap( ~ Location.plot, ncol = 1) +
 ylab("Tree") +
 labs(fill = "Tree\nwas visited") +
 theme_bw() +
 theme(
 axis.ticks.y = element_blank(),
 axis.text.y = element_blank(),
 strip.text = element_text(face="bold", size=rel(1.0)),
 panel.grid.major = element_blank(),
 panel.grid.minor = element_blank(),
 legend.text=element_text(size=7),
 legend.title=element_text(size=9))


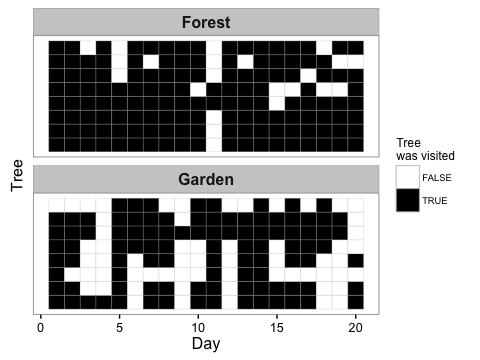


Trees were visited less often in the garden. Some trees were visited less often than others but intratree variation was not strong.

mean(filter(df.Visit, Location == "F")$Visited) # prob Forest tree visited

## [1] 0.8625

mean(filter(df.Visit, Location == "G")$Visited) # prob Garden tree visited

## [1] 0.61875

Fit the GLMM to the data and look for evidence of a location effect.

model <- glmer(cbind(Visited, 1-Visited) ~ Location + (1 | Tree) + (1 | Day),
 family=binomial(link=logit), data = df.Visit)

summary(model)

## Generalized linear mixed model fit by maximum likelihood (Laplace
## Approximation) [glmerMod]
## Family: binomial ( logit )
## Formula: cbind(Visited, 1 - Visited) ~ Location + (1 | Tree) + (1 | Day)
## Data: df.Visit
##
## AIC BIC logLik deviance df.resid
## 329.0 344.1 -160.5 321.0 316
##
## Scaled residuals:
## Min 1Q Median 3Q Max
## -3.0261 -0.5216 0.3439 0.5343 1.7719
##
## Random effects:
## Groups Name Variance Std.Dev.
## Day (Intercept) 0.5697 0.7548
## Tree (Intercept) 0.6931 0.8325
## Number of obs: 320, groups: Day, 20; Tree, 16
##
## Fixed effects:
## Estimate Std. Error z value Pr(>|z|)
## (Intercept) 2.220267 0.001788 1241.5 <2e-16 ***
## LocationG -1.600147 0.001788 -895.1 <2e-16 ***
## ---
## Signif. codes: 0 '***' 0.001 '**' 0.01 '*' 0.05 '.' 0.1 ' ' 1
##
## Correlation of Fixed Effects:
## (Intr)
## LocationG 0.000

drop1(model, test = "Chisq") # LRT test for a location effect

## Single term deletions
##
## Model:
## cbind(Visited, 1 - Visited) ~ Location + (1 | Tree) + (1 | Day)
## Df AIC LRT Pr(Chi)
## <none> 329.02
## Location 1 334.64 7.618 0.005779 **
## ---
## Signif. codes: 0 '***' 0.001 '**' 0.01 '*' 0.05 '.' 0.1 ' ' 1

There is evidence of location differences in visitation rates.

## Create Tree Visitation Figure for Paper (Fig. 4)

# create a jpeg for publication
jpeg("TreeVisits.jpeg", height = 1200, width = 1300, res = 300)
ggplot(df.Visit, aes(x = Day, y = Tree.plot, fill = Visited)) +
 geom_tile(color = "grey") +
 scale_fill_manual(values=c("white", "black")) +
 facet_wrap( ~ Location.plot, ncol = 1) +
 ylab("Tree") +
 labs(fill = "Tree\nwas visited") +
 theme_bw() +
 theme(
 axis.ticks.y = element_blank(),
 axis.text.y = element_blank(),
 strip.text = element_text(face="bold", size=rel(1.0)),
 panel.grid.major = element_blank(),
 panel.grid.minor = element_blank(),
 legend.text=element_text(size=7),
 legend.title=element_text(size=9))
dev.off()

## quartz_off_screen
## 2
